# The E3 ubiquitin ligase skp2 regulates neural differentiation independent from the cell cycle

**DOI:** 10.1186/1749-8104-2-27

**Published:** 2007-12-14

**Authors:** Hector Boix-Perales, Ian Horan, Helen Wise, Horng-Ru Lin, Li-Chiou Chuang, P Renee Yew, Anna Philpott

**Affiliations:** 1 Department of Oncology, University of Cambridge, Hutchison/MRC Research Centre, Addenbrookes Hospital, Hills Road, Cambridge CB2 0XZ, UK; 2 Department of Molecular Medicine, Institute of Biotechnology, University of Texas Health Science Center at San Antonio, San Antonio, Texas, 78245, USA

## Abstract

**Background:**

The SCF^skp2 ^complex is an E3 ubiquitin ligase that is known to target a number of cell cycle regulators, including cyclin-dependent kinase inhibitors, for proteolysis. While its role in regulation of cell division has been well documented, additional functions in differentiation, including in the nervous system, have not been investigated.

**Results:**

Using *Xenopus *as a model system, here we demonstrate that skp2 has an additional role in regulation of differentiation of primary neurons, the first neurons to differentiate in the neural plate. *Xenopus *skp2 shows a dynamic expression pattern in early embryonic neural tissue and depletion of skp2 results in generation of extra primary neurons. In contrast, over-expression of skp2 inhibits neurogenesis in a manner dependent on its ability to act as part of the SCF^skp2 ^complex. Moreover, inhibition of neurogenesis by skp2 occurs upstream of the proneural gene encoding NeuroD and prior to cell cycle exit. We have previously demonstrated that the *Xenopus *cyclin dependent kinase inhibitor Xic1 is essential for primary neurogenesis at an early stage, and before these cells exit the cell cycle. We show that SCF^skp2 ^degrades Xic1 in embryos and this contributes to the ability of skp2 to regulate neurogenesis.

**Conclusion:**

We conclude that the SCF^skp2 ^complex has functions in the control of neuronal differentiation additional to its role in cell cycle regulation. Thus, it is well placed to be a co-ordinating factor regulating both cell proliferation and cell differentiation directly.

## Background

Ubiquitin-mediated proteolysis has recently emerged as a central player in regulating destruction of proteins controlling cell fate determination, cell proliferation and cell differentiation [1,2]. Indeed, coordinating stability of proteins involved in these three processes is an efficient way for cells to decide to divide or to undergo differentiation.

Polyubiquitination, which targets proteins for destruction, is brought about by a multi-enzyme cascade, typically involving E1 ubiquitin-activating enzymes, E2 ubiquitin-conjugating enzymes and E3 ubiquitin ligases [3]. Specificity for particular substrates resides in the E3 subunit, which is itself often a multi-subunit complex. The largest group of E3 ligases is the ring-finger domain family and within this family several so called SCF E3 ligases are known to target proteins important for maintenance of the balance between proliferation and differentiation [4-6]. One of the best studied ligases in this family is SCF^skp2^, which is composed of a cullin-1 scaffold, bound directly to the Ring finger protein Rbx1 and to Skp1 [4-9]. Specificity for ubiquitination resides in the skp2 subunit, which associates with Skp1 via its F box.

SCF^skp2 ^has been shown to target a growing number of proteins important in cell cycle progression for ubiquitin-mediated proteolysis. These include the transcription factors E2F1 [10] and c-myc [11,12], and the cdk inhibitors (cdkis) p21 Cip1 [13,14] and p27Kip1 [15], although the physiological relevance of some of these potential substrates has been questioned.

Importantly, targets of SCF^skp2^, of which p27Kip1 is the most studied, must generally be phosphorylated in a cell cycle dependent manner to be targeted by skp2 for polyubiquitination and destruction [16-18]. *Xenopus *skp2 has recently been cloned and has been shown to target the *Xenopus *cdki Xic1 [19,20] for degradation *in vitro *and in egg extracts [21]. However, in this case Xic1 appears not to require phosphorylation for targeting.

We have previously showed that Xic1 has an essential role in differentiation of primary neurons, the first neurons to differentiate in the neural plate in *Xenopus *[22]. Neural differentiation in this system is driven by the neurogenic transcription factor, Neurogenin (XNgnr1), which is expressed in scattered neural precursors in the neural plate arranged in three stripes lateral to the midline [23]. XNgnr1 transcriptionally upregulates another basic helix-loop-helix (bHLH) transcription factor NeuroD, whose expression coincides with cell cycle exit and terminal neural differentiation [24]. Interestingly, we have shown that Xic1 is not required at these late stages downstream of NeuroD for cell cycle exit or differentiation. Instead, Xic1 acts at an early stage of neurogenesis, in parallel with XNgnr1, where it appears to stabilise the XNgnr1 protein [22]. Moreover, this function is separable from the ability of Xic1 to inhibit overall cdk kinase activity [22]. Thus, Xic1 has distinct roles in cell cycle regulation and differentiation, making it an appealing candidate for a protein that coordinates these two functions.

Skp2, by regulating the stability of cdkis as well as additional targets such as c-myc and E2F1, is also well placed to play a role in coordinating proliferation and differentiation. A skp2 null mouse has been generated, which although smaller than wild type, is phenotypically grossly normal despite an increase in polyploidy, multiple centrosomes and apoptosis [15]. A skp2-/- p27Kip1-/- mouse has also been generated [25], which appears similar but not identical to the p27Kip1-/- mice, indicating that, while p27Kip1 may be its main target, SCF^skp2 ^may ubiquitinate other important substrates. In mice, however, the presence of three cdkis and other redundancies makes analysis of the role of skp2 in proliferation and differentiation during development problematic.

Importantly, targets of SCF^skp2^, of which p27Kip1 is the most studied, must generally be phosphorylated in a cell cycle dependent manner to be targeted by skp2 for polyubiquitination and destruction [16-18]. *Xenopus *skp2 has recently been cloned and has been shown to target the *Xenopus *cdki Xic1 [19,20] for ubiquitination *in vitro *and for degradation in egg extracts [21]. However, in this case Xic1 appears not to require phosphorylation for targeting.

We find that *Xenopus *skp2 (skp2) shows dynamic expression in the neural plate and neural tube, indicating that it is well placed to regulate neural differentiation in a stage-specific manner. Loss of skp2 protein after morpholino injection has no effect on the cell cycle but results in differentiation of extra primary neurons, while over-expression of skp2 inhibits neurogenesis and this depends on the presence of an F box. Skp2 can degrade Xic1 *in vivo *and this can account for some, but not all, of skp2's ability to regulate primary neurogenesis. Therefore, skp2 is well placed to directly regulate the balance between proliferation and differentiation, in addition to a potential role in cell cycle control.

## Results

**Figure 1:**
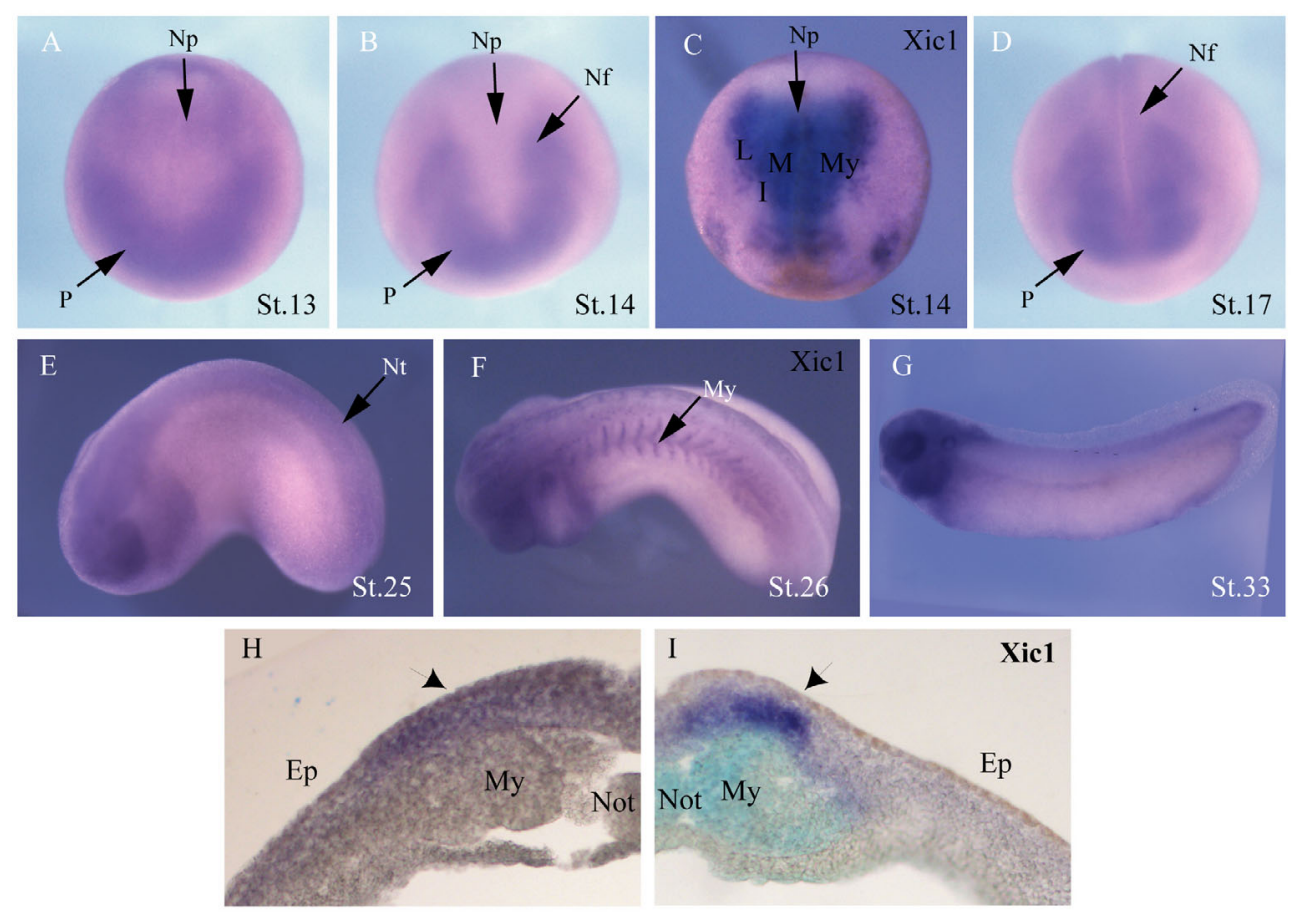
Expression of skp2 and Xic1. Whole-mount *in situ *hybridisation at indicated stages show expression of **(a,b,d,e,g) **skp2 and **(c,f) **Xic1. (a,c) Dorsal view with anterior toward the bottom. (b,d) Anterior view with dorsal toward the top. (e-g) Lateral view with anterior to the left. **(h,i) **Expression of skp2 and Xic1, respectively, in a vibratome section of stage 16 embryos; primary neurons are indicated with black arrows. Ep, epidermis; I, intermediate stripe; L, lateral stripe; M, medial stripe; My, myotome; Nf, neural folds; Not, notochord; Np, neural plate; Nt, neural tube; P, placodes.

Skp2 is expressed at significant but low levels before the mid-blastula transition in *Xenopus *[21]. However, after the mid-blastula transition, levels rise and remain constant through neural plate stages, when primary neurogenesis is occurring. To determine the spatial expression pattern of skp2 during development, *in situ *hybridisation was performed on embryos of different stages (Figure 1). skp2 RNA is expressed broadly early in development (data not shown) but post-gastrulation by stage 13, it is found concentrated in anterior placodes and extending posteriorly, lateral to the edges of the neural plate (Figure 1a). By stage 14, while remaining in the placode and anterior neural folds, skp2 message is excluded from the neural plate proximal to the midline and myotome (Figure 1b), although it is detectable in lateral neural plate regions. For comparison, Xic1 expression was investigated in stage 14 embryos (Figure 1c). Xic1 is strongly expressed in lateral, intermediate and medial stripes corresponding to the positions where primary neurons will differentiate, as well as showing strong staining in the underlying myotome [26]. Thus, at this stage, skp2 expression in the lateral folds overlaps with that of Xic1 in the forming lateral stripe of primary neurons.

We also investigated the expression of skp2 relative to Xic1 in the late neural plate in embryos that had undergone *in situ *hybridisation to detect expression of the individual genes, which were subsequently sectioned (Figure 1h,i). As expected, Xic1 is expressed in deep layer cells, in addition to staining in the underlying myotome and notochord (which can be distinguished by light blue staining due to poor penetration by the BM purple substrate; see Materials and methods). Skp2 staining, by contrast, is weaker but is present in both the superficial and deep layers of the neural plate in a region overlapping Xic1 expression.

By stage 17, when the neural tube begins to close, skp2 message is found posteriorly in the neural folds, as well as remaining in the placodes, eye field and brain regions (Figure 1d). Primary neurons only form from the deep layer of the neural plate while superficial layer cells remain undifferentiated at this stage [27]. By tailbud stage 25, skp2 message is expressed in the neural tube, eye and branchial arches (Figure 1e). At a similar stage (stage 26; Figure 1f), Xic1 is also expressed in the neural tube, eye and branchial arches but additional staining is seen in the myotome where skp2 is undetectable. By stage 33, skp2 expression remains strongest in the head (Figure 1g).

Thus, skp2 is expressed at the right time and in the right place to be playing a role in primary neurogenesis. Interestingly, at early neural plate stages skp2 RNA is excluded from the medial areas expressing the potential target Xic1 (compare Figure 1b and 1c), although overlap is seen in the lateral neural folds (Figure 1h,i). However, as neurulation proceeds, skp2 expression expands to the mid-line (Figure 1d). Therefore, skp2 expression is dynamic and is consistent with a stage dependent role in regulation of Xic1 protein, an *in vitro *target in primary neurons [21]. As Xic1 is essential for primary neurogenesis, skp2 is also well placed for regulating this process. We have investigated this further.

**Figure 2:**
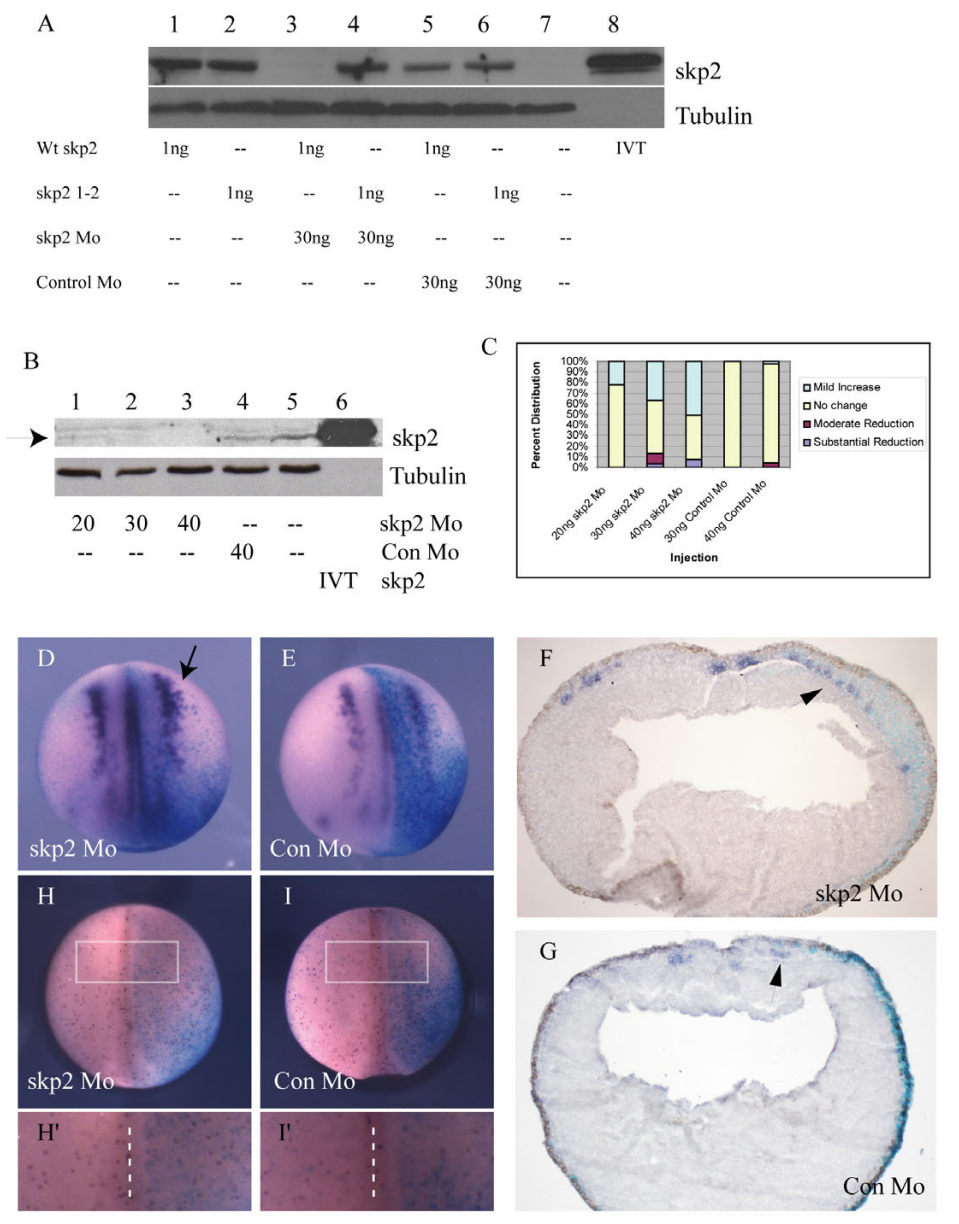
Loss of skp2 protein promotes primary neurogenesis. **(a) **Embryos were injected with wild-type (Wt) skp2, skp2 1–2 alone or in combination with skp2 Mo or Con Mo as indicated. Protein (30 μg) from a stage 15 embryo was western blotted to determine skp2 levels; tubulin was used as a loading control. **(b) **Western blot for endogenous skp2 protein levels on stage 15 embryos that were injected with 20 ng, 30 ng, or 40 ng skp2 Mo or 40 ng Con Mo at the one cell stage, arrow to skp2 protein band. *In vitro *translated (IVT) skp2 protein is run in lane 6, and alpha-tubulin was used as a loading control. **(c) **The percentages of embryos with mild increase, no change, or moderate or substantial reduction of nßt positive cells on the injected side relative to the uninjected side for 20 ng, 30 ng, and 40 ng skp2 Mo, or 30 ng and 40 ng Con Mo (see Additional file 1 for photographs of representative embryos). **(d,e) **Embryos were injected with 30 ng skp2 Mo (d) or 30 ng Con Mo (e) in one blastomere at the two cell stage, along with ßgal mRNA as a lineage tracer, and analyzed for nßt mRNA expression at stage 15 ((d) arrow to show expansion of primary neurons). The view is dorsal with injected side to the right. **(f,g) ***In situ *hybridisation sections, which are transverse across the centre of the embryo, with injected side to the right (f) Section of an early neurula embryo injected with 30 ng skp2 Mo, indicating nßt upregulation by skp2 protein depletion. (g) Section of a mid neurula embryo injected with 30 ng Con Mo showing no difference in nßt distribution. Arrows (f, g) denoting staining of nßt in primary neurons. **(h,i) **Whole mount stage 15 embryos immunostained (red) to detect pH3 after injection of 30 ng skp2 Mo (h) or 30 ng Con Mo (i) in one blastomere at the two cell stage. ßgal mRNA was co-injected and X-Gal staining (blue) was performed to reveal injected side. Dorsal views with injected side to the right. **(h',i') **Detail of pH3 cells on the injected side relative to the uninjected side of representative embryos (boxed area in (h,i), dashed line is dorsal mid-line separating injected and uninjected halves).

To determine whether skp2 may play a role in regulation of primary neurogenesis, we designed a skp2 morpholino (skp2 Mo) to inhibit skp2 protein expression, along with a matched control morpholino (Con Mo) that has five base changes, so would be unable to bind to skp2 message. As expected, skp2 Mo but not Con Mo was able to prevent translation of co-injected skp2 message (Figure 2a), although skp2Mo is unable to target a modified skp2 that is missing the first six base-pairs after the initiator ATG (skp2 1–2). We then injected increasing amounts of skp2 Mo into two of two cells and performed western blot analysis to look at the level of endogenous skp2 protein (Figure 2b). While endogenous skp2 is expressed at low levels, probably as it is an unstable protein [28], it is nevertheless clearly detected in both uninjected embryos and those injected with 40 ng Con Mo (Figure 2b, lanes 4 and 5). In contrast, 20 ng of skp2 Mo substantially inhibited expression of skp2 compared to uninjected or Con Mo injected embryos (Figure 2b, lane 1), and at 30 ng and 40 ng of skp2 Mo, skp2 protein was undetectable (Figure 2b, lanes 2 and 3). Thus, skp2 Mo specifically blocks translation of skp2 message, resulting in an absence of detectable skp2 protein, while a matched control morpholino has no effect.

We then investigated the effect of loss of skp2 on differentiation of primary neurons, by injecting skp2 Mo or Con Mo into one cell of a two-cell embryo along with β-galactosidase (ßgal) RNA as a lineage tracer. As the first plane of cleavage should bisect the embryo along the left/right axis, the injected and uninjected sides of the embryo can be compared. Embryos were allowed to develop to stage 15, fixed and then *in situ *hybridisation was performed to determine neural β-tubulin (nßt) expression, which is expressed in differentiated primary neurons (Figure 2c–g). Embryos were scored according to the number of neurons on the injected versus the uninjected side. Examples of representative embryos displaying the phenotypes described (Figure 2c) are given (Additional file 1). Skp2 Mo injection resulted in a subtle but reproducible increase in the number of primary neurons on the injected side versus the uninjected side of the embryo, generally proximal to or within the usual stripe of primary neurogenesis (Figure 2c, d, f; 20 ng, n = 120 embryos; 30 ng, n = 123 embryos; 40 ng, n = 100 embryos). This effect was dependent on the dose of skp2 Mo injected; the higher the dose of skp2 Mo, the greater the percentage of embryos showing an increase in neuron number (Figure 2c). A very small proportion of embryos injected with skp2 Mo at the highest doses showed a reduction of neurons. However, this was not strictly dose dependent and a similar, albeit slightly smaller, effect was also seen with the highest dose of Con Mo (30 ng, n = 73; 40 ng, n = 108), so it is unlikely to be a specific effect of loss of skp2 protein.

The increase in the number of primary neurons on the injected side was subtle and somewhat variable in wholemount embryos. To look more closely, stage 15 embryos were sectioned and analyzed by *in situ *hybridisation for nßt expression. Extra primary neurons were clearly seen after skp2Mo injection on the injected side only, and these were confined, as usual, to the deep layer of cells of the neuroectoderm (Figure 2f, arrow). Interestingly, we usually saw an increase in primary neurons lateral to or within the lateral stripe rather than at the mid-line. Cells in the lateral stripe will go on to form the sensory neurons and this is the region of neural plate-stage embryos where *in situ *hybridisation staining for both Xic1 and skp2 overlap (Figure 1b, c, h, i).

Thus, loss of skp2 protein leads to a moderate but dose-dependent and significant increase in the number of primary neurons. Notably extra neurons form generally dorso-laterally and near to or within the established stripes in the deep layer of the neural plate but not ectopically significantly ventrally (Figure 2d, f). A similar effect is seen on over-expression of the skp2 target, Xic1 [22].

SCF^skp2 ^degrades cdkis [10,13,14,29-32]. Therefore, loss of skp2 might be expected to result in a slowing of the cell cycle, and indeed skp2 knockout mice are small and their cells display a reduced growth rate [15]. As proliferation is thought to be incompatible with differentiation in neurons, it is therefore possible that skp2 Mo is promoting neurogenesis by inducing cell cycle exit in the neural plate. To investigate this possibility, we injected skp2 Mo or Con Mo into one cell of a two cell embryo and allowed these embryos to develop to neural plate stage before staining for the mitotic marker phosphohistone H3 (pH3) [33] (Figure 2h, i). Numbers of pH3-expressing cells were counted in a fixed area of the neural plate on injected and uninjected sides and compared (Figure 2h', i'). There was no significant difference in the number of mitotic cells after injection of either skp2 Mo or Con Mo (n = 31 embryos and n = 38 embryos, respectively). Therefore, loss of skp2 does not promote neural differentiation by forcing cell cycle exit. A thickening of the epidermis was sometimes seen in sections (Figure 2f), indicating some alteration in neuroectodermal architecture. However, our pH3 analysis indicates this is not a result of increased proliferation of neuroectodermal cells.

**Figure 3:**
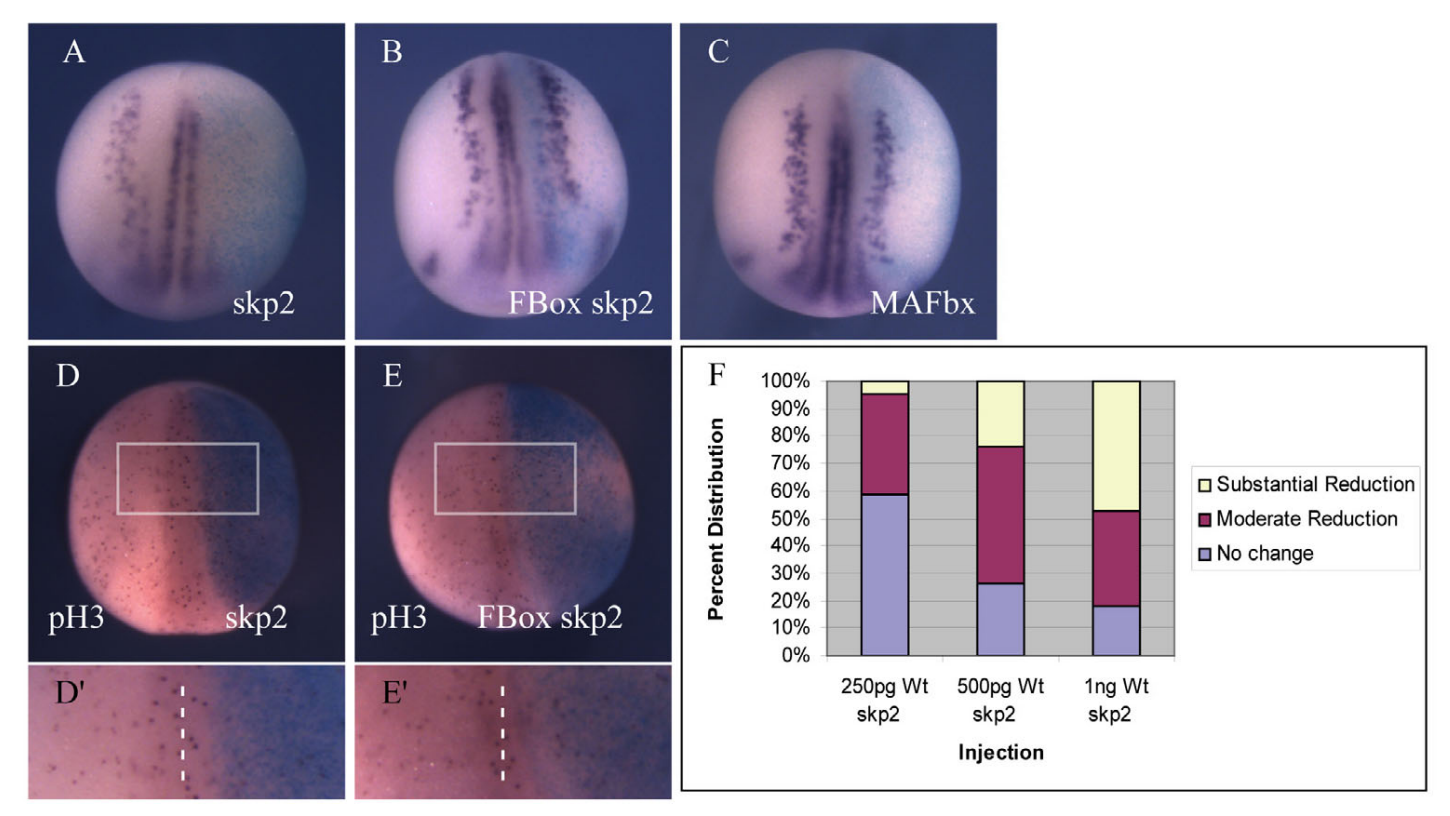
Over-expression of skp2 blocks primary neurogenesis and reduces cell proliferation. **(a-e) **Embryos were injected with 1 ng wild-type (Wt) skp2 (a,d), 1 ng FBox skp2 (b,e), or 1 ng Wt MAFbx (c) mRNA, along with ßgal mRNA as a lineage tracer, in one blastomere at the two cell stage. Embryos were analyzed for expression of nßt mRNA (a-c) at stage 15. Dorsal views with injected side to the right. (d,e) Whole mount stage 15 embryos immunostained (red) to detect pH3 after injection of 1 ng Wt skp2 (d), or 1 ng FBox skp2 (e) mRNA, along with ßgal mRNA as a lineage tracer, in one blastomere at the two cell stage. Dorsal views with injected side to the right. (d',e') Detail of pH3-positive cells on the injected side relative to the uninjected side (boxed area in (d,e), dashed line is dorsal mid-line separating injected and uninjected halves). **(f) **Embryos were injected with 250 pg, 500 pg or 1 ng Wt skp2 mRNA (n = 41, 83, 87 embryos, respectively) in one blastomere at the two cell stage. Embryos were analyzed for expression of nßt mRNA at stage 15. Data shown in (f) are the percentages of embryos with no change, or moderate or substantial reduction of nßt positive cells on the injected side relative to the uninjected side for each injection (see Additional file 1 for photographs of representative embryos).

While skp2 targets need to be phosphorylated prior to ubiquitination, the absolute amount of skp2 may also be important in determining target degradation; levels of skp2 protein itself are regulated in a cell cycle dependent manner [25,34] while its levels are elevated in a number of human tumours [3,35-54]. Therefore, we next determined the effect of over-expression of skp2 RNA on differentiation of primary neurons (Figure 3a–c,f). As controls, we used the mutant of skp2 that has had the F box Skp1 binding domain deleted (F box skp2), so is impaired in its ability to target substrates for destruction [21]. This F Box-deleted form of skp2 has been shown to be unable to target Xic1 for destruction *in vitro *[21]. As an additional control, we injected RNA encoding *Xenopus *MAFbx. MAFbx is a related ring finger E3 ligase that can ubquitinate MyoD in mammals [55] and, therefore, would not be expected to affect primary neurogenesis.

To investigate the effect of over-expression of skp2, RNAs were injected into one cell of two cell embryos along with ßgal RNA, and then embryos were allowed to develop until stage 15. *In situ *hybridisation was subsequently performed to detect nßt expression; 84% of embryos (n = 359) injected with wild-type skp2 showed reduced nßt expression on the injected side (Figure 3a). In contrast, neither F box skp2 (Figure 3b) nor MAFbx (Figure 3c) affected nßt staining (100% n = 65 and 92% n = 49 unaffected, respectively). Thus, over-expression of skp2 inhibits neuronal differentiation and this is dependent on the ability of skp2 to interact with the SCF complex and, hence, target substrates for ubiquitin-mediated proteolysis. The effect of skp2 over-expression is dose-dependent; embryos injected with 250 pg skp2 mRNA showed only a modest decrease in neurons but this effect was progressively more pronounced on increasing skp2 mRNA, up to 1 ng, where more than 80% of embryos were affected (Figure 3f).

Elevated levels of skp2 correlate with cancer where cells proliferate inappropriately [38,56]. Moreover, skp2 over-expression alone induces quiescent fibroblasts to replicate their DNA in low serum [30]. Such an increase in cell proliferation might be expected to inhibit neural differentiation and, indeed, over-expression of cyclin A2/cdk2 alone in *Xenopus *embryos inhibits primary neurogenesis [57]. Thus, we investigated whether skp2 over-expression alone could promote proliferation in early *Xenopus *embryos by injection of skp2 mRNA into one cell of a two cell embryo followed by analysis of pH3 staining in the neural plate. Unexpectedly, skp2 over-expression led to a 24% decrease in pH3-expressing cells on the injected side (*p *< 0.001, n = 27 embryos; Figure 3d). Moreover, the F box mutant of skp2 also produced an 18% decrease in pH3 cells (*p *< 0.001, n = 17 embryos; Figure 3e). This indicates that effects on proliferation are modest and are not due to the specific targeting of skp2-dependent substrates for ubiquitin-mediated proteolysis, and additionally cannot explain the ability of wild-type skp2 to inhibit neurogenesis.

Depletion of skp2 promotes primary neurogenesis (Figure 2) while over-expression of skp2 inhibits neuron formation (Figure 3). To demonstrate the specificity of these effects, we co-injected skp2 Mo or Con Mo along with RNA encoding skp2 missing the first six base-pairs after the ATG start site, which is not targeted by the skp2 Mo. Western blotting of protein lysates from stage 15 embryos revealed that skp2 Mo knocks down exogenous skp2 protein effectively, but is unable to reduce levels of this non-targeted skp2 protein (Figure 2a).

**Figure 4:**
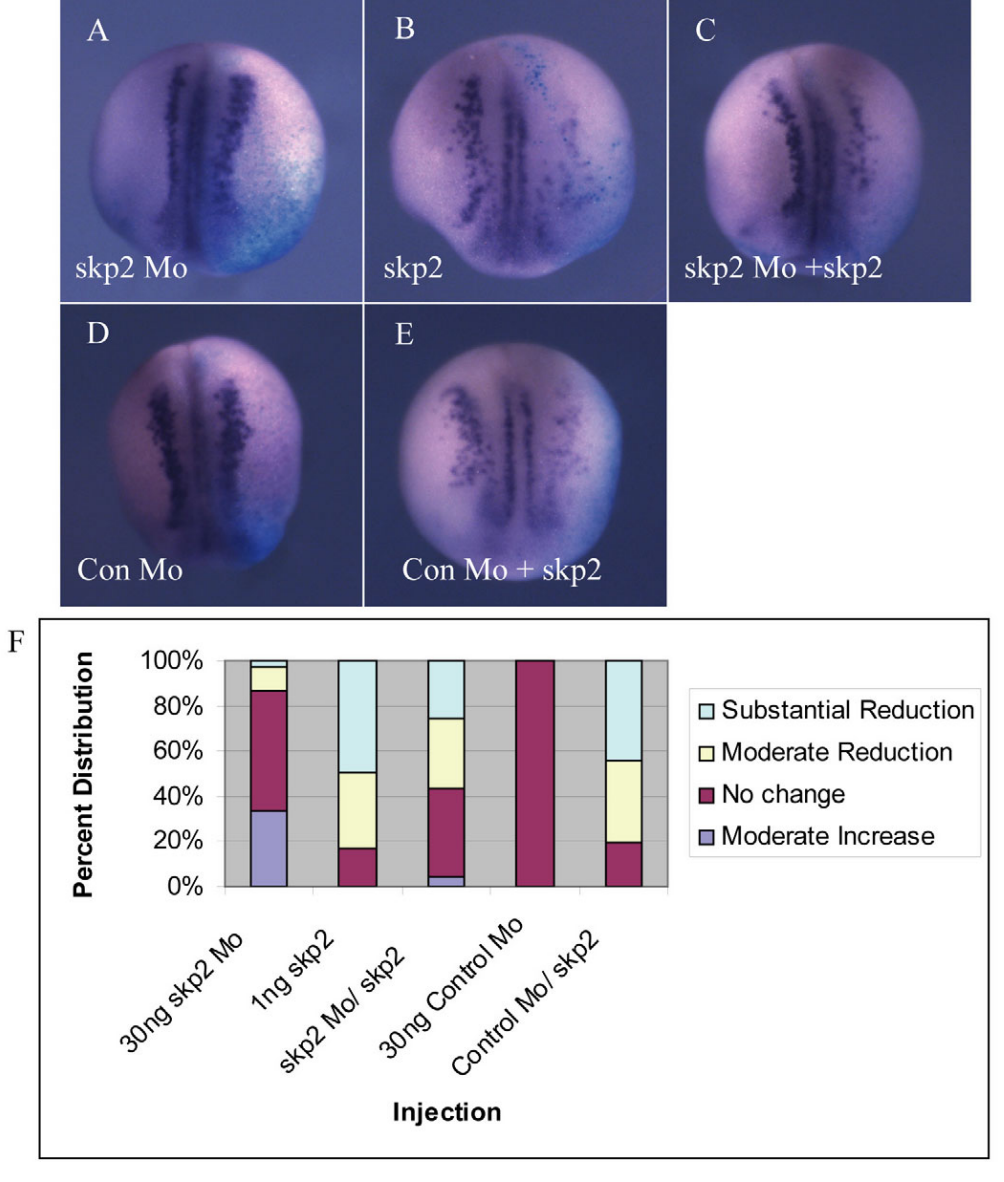
Over-expression of skp2 counteracts the effect of skp2 Mo on neural differentiation. **(a-e) **Embryos were injected with 30 ng skp2 Mo (a), 30 ng Con Mo (d), or 1 ng skp2 (missing first two amino acids) mRNA (b) alone, or together as labelled (c,e), in one blastomere at the two cell stage. ßgal mRNA was co-injected as a lineage tracer. Embryos were analyzed for expression of nßt mRNA at stage 15. Dorsal views with injected side to the right. **(f) **Percentages of embryos with moderate increase, no change, or moderate or substantial reduction of nßt positive cells on the injected side relative to the uninjected side for each injection.

Injection of skp2 Mo resulted in a modest increase in nßt-expressing cells (Figure 4a, f; 33%, n = 51 embryos), while injection of non-targeted skp2 alone resulted in 80% of embryos showing a decrease in neurogenesis (Figure 4b, f, n = 79 embryos). Co-injection of skp2 Mo and skp2 mRNA resulted in an intermediate phenotype (Figure 4c, f). Thus, the absolute level of skp2 protein is crucial for regulating the number of primary neurons forming in the neural plate.

**Figure 5:**
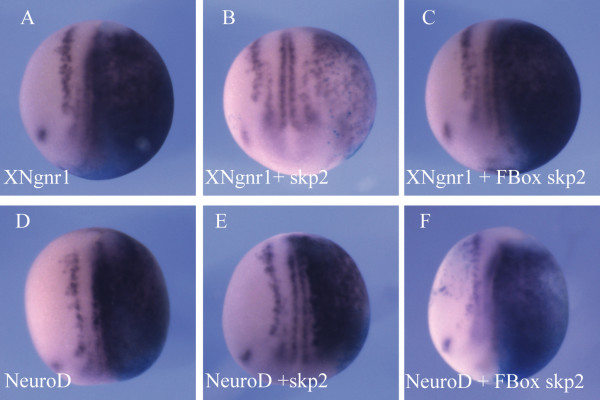
skp2 inhibits primary neurogenesis between XNgnr1 and NeuroD. **(a-f) **Embryos were injected with XNgnr1 (a) or NeuroD (d) mRNA alone, or together with Wt skp2 (b,e) or FBox skp2 (c,f) mRNA, in one blastomere at the two cell stage. ßgal mRNA was co-injected as a lineage tracer. Embryos were analyzed for expression of nßt mRNA at stage 15. Dorsal views with injected side to the right.

Cell cycle exit of primary neurons is thought to occur downstream of NeuroD [58]. To determine where skp2 acts in the pathway of neurogenesis, we co-injected wild-type or F Box skp2 mRNA along with XNgnr1 mRNA, the earliest acting proneural gene [23], or mRNA for its downstream target NeuroD [24] (Figure 5). Embryos were fixed at stage 15 and stained for nßt expression. Over-expression of XNgnr1 induces extensive ectopic neurons on the injected side (Figure 5a; 100%, n = 152 embryos). However, when skp2 is co-injected, 86% of embryos showed reduced neuronal staining compared to XNgnr1 alone (Figure 5b; n = 113 embryos). In contrast, F Box skp2 had no effect on XNgnr1's ability to induce neurons (Figure 5c; 100%, n = 27 embryos). Over-expression of the downstream bHLH neurogenic transcription factor NeuroD also induces substantial ectopic neurogenesis (Figure 5d; 100%, n = 70 embryos), but this was not inhibited by co-injection of either wild-type or F Box skp2 (Figure 5e, f; both 100%, n = 80 and n = 35 embryos, respectively). Therefore, over-expression of skp2 inhibits neurogenesis upstream of NeuroD, but in parallel with, or downstream of, XNgnr1. As primary neurons are thought to exit the cell cycle downstream of NeuroD expression [58], taken together with results from pH3 staining (Figures 2h and 3d), skp2 appears not to regulate primary neurogenesis by regulating cell cycle progression and exit.

Skp2 has been demonstrated in the mammalian systems to degrade several substrates known for their roles in regulating proliferation and/or differentiation, including p27Kip1, p21Cip1, c-myc, E2F-1 and p107 [10,13,14,29-31,59]. Xic1, the *Xenopus *homologue of p27Kip1 and p21Cip1, is also targeted by skp2 for ubiquitin-mediated proteolysis [21]. We have previously shown that Xic1 plays an essential role in primary neurogenesis in parallel with XNgnr1 and prior to cell cycle exit [22]. As over-expression of skp2 inhibits neurogenesis at this stage, skp2 may regulate primary neurogenesis by controlling the levels of Xic1 protein. We have investigated this possibility further.

**Figure 6:**
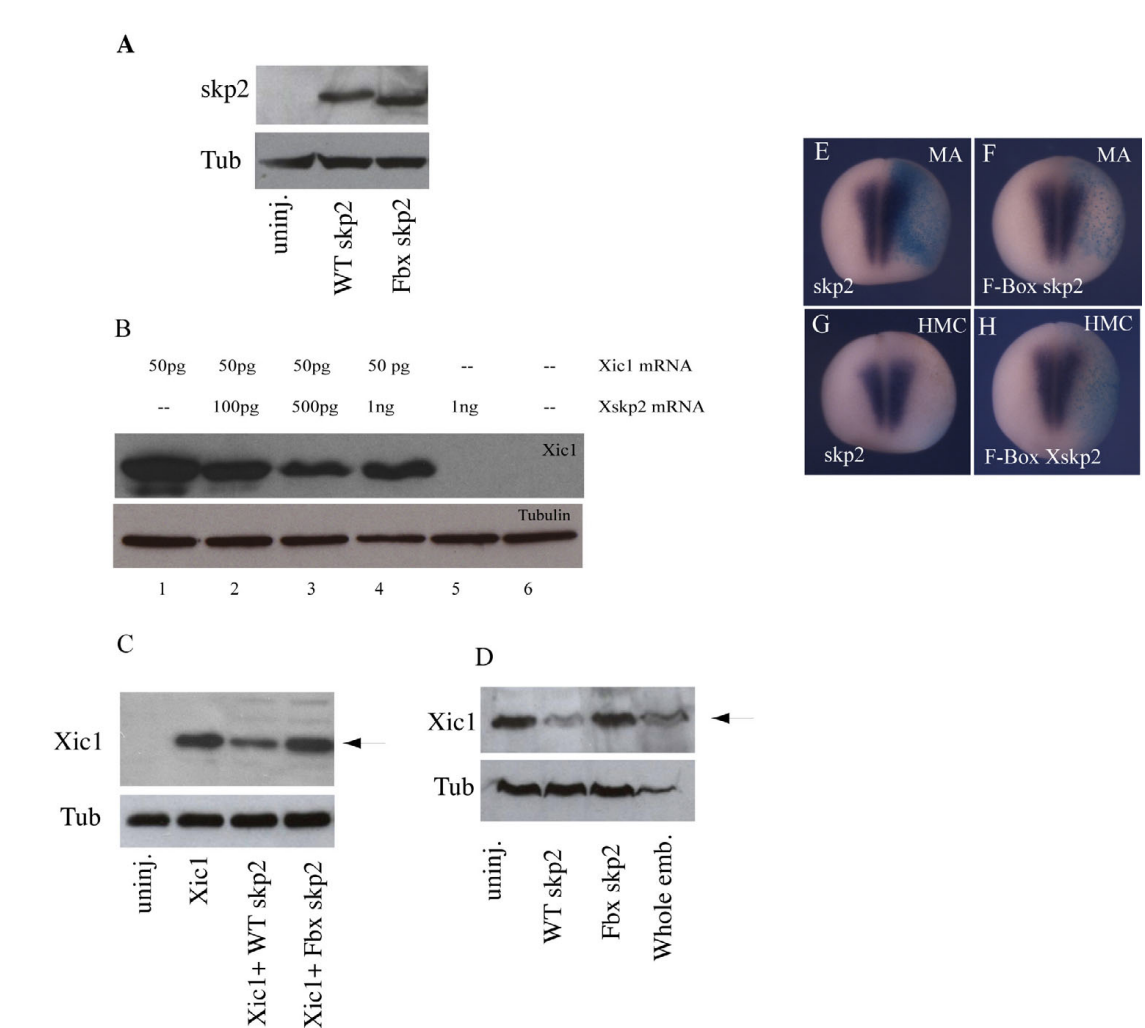
Over-expression of skp2 can degrade p27Xic1. **(a) **Western blot for embryos at stage 9, either uninjected or injected with 1 ng mRNA encoding wild-type (WT) skp2 or F box skp2, as labelled; tubulin demonstrates equal loading. **(b) **Western blot for Xic1 levels in embryos injected with 50 pg Xic1 mRNA and increasing doses (100 pg to 1 ng) of skp2 mRNA at the one cell stage. Embryos were harvested at stage 13; tubulin demonstrates equal loading. **(c) **Western blot for Xic1 levels in embryos harvested at stage 7 that were injected with 1 ng WT skp2 or 1 ng FBox skp2 mRNA at the one cell stage along with 50 pg Xic1 mRNA; tubulin demonstrates equal loading. **(d) **Western blot for Xic1 levels in neural plates harvested at stage 16 from embryos that were injected with 1 ng WT skp2 or 1 ng FBox skp2 mRNA into the animal pole of a fertilised egg. Tubulin demonstrates equal loading. **(e-h) **Embryos were injected with 1 ng WT skp2 (e,g) or 1 ng FBox skp2 (f,h) mRNA, along with ßgal mRNA as a lineage tracer, in one blastomere at the two cell stage. Embryos were analyzed for expression of muscle actin (e,f) or heavy chain myosin expression (g,h) at stage 15. Dorsal views with injected side to the right.

Skp2 can target Xic1 for ubiquitination in a fully reconstituted biochemical assay and can target it for destruction *in vitro *using extracts of *Xenopus *eggs [21]. We wished to determine whether over-expressed skp2 could promote Xic1 degradation. Firstly, we compared the level of expression of wild-type skp2 and the F-box-deleted mutant skp2 (Figure 6a). After injection into fertilised eggs, embryos were harvested at stage 9 and western blotting performed to detect over-expressed skp2 protein. Both wild-type and F box skp2 were detected. To determine whether over-expressed skp2 can target Xic1 for degradation in developing embryos, we injected increasing amounts of skp2 RNA up to 1 ng with or without RNA encoding Xic1, and then performed western blot analysis to look at Xic1 levels at stage 13 (Figure 6b). When injected alone, Xic1 protein accumulated to considerable levels (Figure 6b, lane 1). Levels of Xic1 protein dropped on co-injection with skp2, showing a maximal decrease at 500 pg co-injected skp2 message (Figure 6b, lanes 2–4). Therefore, while increasing skp2 levels did promote Xic1 destruction, this was not a linear relationship at high levels of skp2, perhaps resulting from saturation of the rest of the SCF^skp2 ^machinery required for skp2 to act catalytically. Skp2-mediated degradation of Xic1 is dependent on an intact F Box (Figure 6c).

Skp2 message is most strongly expressed in the neural plate, underlying myotome and the notochord at stage 15. Xic1 is also transiently expressed in scattered cells in the epidermis at around stages 12–13 [22] so protein may still be present in the skin of the embryo as well as these other tissues. To determine whether skp2 can target endogenous Xic1 for degradation in the neural plate, embryos were injected with wild-type or F Box skp2 dorsally along with green fluorescent protein as a lineage tracer. The dorsal region encompassing the neural plate and anterior neural folds of stage 16 embryos expressing green fluorescent protein in this area were dissected out, and the levels of Xic1 protein compared by western blotting. Over-expression of wild-type skp2 significantly decreased the level of endogenous Xic1 protein compared to neural plates from uninjected embryos (Figure 6d). In comparison to wildtype skp2, Fbox skp2 had no effect on the level of endogenous Xic1 protein (Figure 6d), consistent with its impaired ability to target Xic1 to the SCF complex.

Interestingly, over-expression of skp2 did not result in complete degradation of either endogenous Xic1 (Figure 6d) or Xic1 translated from co-injected cDNA (Figure 6b, c). As well as expression in the epidermis, primary neurons and neuronal placodes, Xic1 becomes increasingly strongly expressed in the myotome as neural plate stages progress (Figure 1). Xic1 is absolutely required for myotomal muscle differentiation in *Xenopus *[26], so if it can be degraded by skp2 in this tissue we would expect skp2 over-expression to inhibit myogenesis. Hence, we looked to see whether wild-type skp2, but not Fbox skp2, could inhibit muscle differentiation. Interestingly, and unlike differentiation of primary neurons, over-expression of skp2 did not inhibit early or late muscle differentiation (Figure 6e–h), indicating that Xic1 protein cannot be targeted for destruction in this tissue. This would account for our observation that only a proportion of Xic1 can be degraded by skp2 over-expression; Xic1 protein in the myotome would remain stable.

**Figure 7:**
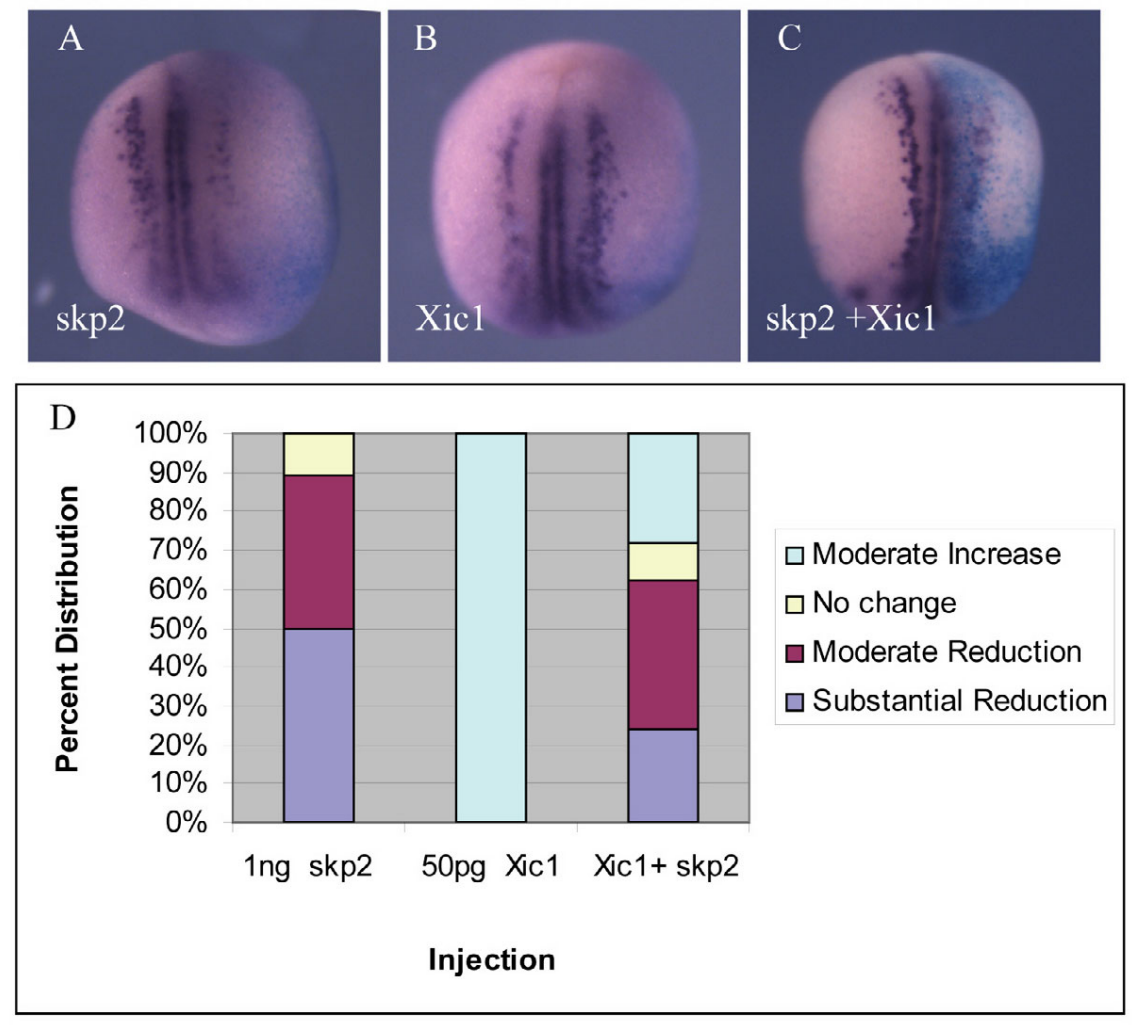
The effect of skp2 over-expression on neural differentiation is partially rescued by extra Xic1. **(a-c) **Embryos were injected with wild-type skp2 (a) or wild-type Xic1 (b) mRNA alone, or together (c), in one blastomere at the two cell stage. ßgal mRNA was co-injected as a lineage tracer. Embryos were analyzed for expression of nßt mRNA at stage 15. Dorsal views with injected side to the right. **(d) **Percentages of embryos with mild increase, no change, or moderate or substantial reduction of nßt-positive cells on the injected side relative to the uninjected side (see Additional file 1 for photographs of representative embryos).

Xic1 over-expression promotes primary neurogenesis [22]. To investigate further whether Xic1 is a target for skp2 in the neural plate during neurogenesis *in vivo*, we injected skp2, Xic1 or both mRNAs into one cell of two cell embryos, allowed embryos to develop to stage 15 and then looked for nßt expression (Figure 7). On injection of skp2, 50% of embryos showed a substantial reduction in nßt, often resulting in an absence of neurons on the injected side, while a further 39% showed a moderate reduction (n = 155). In contrast, 100% of embryos injected with Xic1 showed a moderate increase in the numbers of neurons (n = 131). When both mRNAs were co-injected, we saw a selection of intermediate phenotypes; 24% showed a substantial reduction in neurons, 38% a moderate reduction, 10% no difference, and 28% a moderate increase (Figure 7d).

Thus, over-expression of Xic1 can partially rescue the effect of skp2 over-expression. However, it is of note that, after mRNA injection, Xic1 protein accumulates to levels considerably above endogenous (for example, compare lanes 1, 4 and 5 in Figure 6b), yet this is not enough to fully reverse the effects of skp2 over-expression on inhibition of neurogenesis. Thus, we consider it likely that skp2 is regulating levels of both Xic1 and other factors required for primary neurogenesis. These potentially novel substrates remain to be identified.

## Discussion

Ubiquitin-mediated proteolysis has recently emerged as a central regulator of numerous cellular pathways, although the role of E3 ubiquitin ligases in developmental processes has been poorly studied. We have investigated the role in early *Xenopus *development of skp2, a component of the E3 ligase SCF^skp2 ^previously shown to be important for cell cycle regulation.

*In situ *hybridisation demonstrates that the developmental expression of skp2 is dynamic and is consistent with skp2 playing a role in neurogenesis (Figure 1). At early neural plate stages, skp2 is expressed in anterior regions corresponding to the neuronal placodes, eye field and brain, and the lateral neural folds where neuronal precursors are abundant, but is excluded from medial regions of the posterior neural plate. Expression in the neural plate at this time overlaps at least partially with that of its potential target, Xic1, although while Xic1 expression is confined to the deep layer of cells in the neural plate, skp2 mRNA is found in both the deep and superficial layers (Figure 1h). As neurulation progresses, skp2 becomes upregulated throughout the neural tube, although remaining most prominent in the anterior regions, including in the eye and in the migrating neural crest of the branchial arches. At later stages skp2 staining is lost in the posterior of the embryo, although it remains high in the head. Thus, the timing and localisation of skp2 message are consistent with it playing a role in regulation of Xic1 stability and primary neurogenesis. It is of note that Xic1 is also strongly expressed in the myotome (Figure 1c, i), where it is required for muscle differentiation, but skp2 is not expressed in this tissue (Figure 1h), indicating that any role of skp2 in regulating Xic1 levels may be confined to neural tissue.

SCF^skp2 ^has a known role in cell cycle progression, which it effects by controlling the levels of a number of key cell cycle regulators, including cdkis, E2F and c-myc [10-14,29-32]. We investigated the effect of up- or down-regulation of skp2 levels on cell cycling by measuring pH3 staining, a specific marker for mitotic cells [33]. Surprisingly and in contrast to the mouse, loss of skp2 protein had no effect on cell proliferation in early *Xenopus *embryos (Figure 2f, h). One of the major points of regulation for progression through the cell cycle is called the restriction point, found in G1. Passage through the restriction point and, thus, commitment to cell cycling is controlled by phosphorylation of the retinoblastoma (pRb) protein. This results in release of the transcription factor E2F, which in turn drives transcription of genes required for S phase progression (for a review, see [60]). pRb phosphorylation is brought about by cdks, which are in turn inhibited by cdkis. Both cdkis and E2F are targets for skp2-mediated ubiquitination and destruction. Loss of skp2 in mammalian cells results in cdki accumulation [25], which will cause pRb to be maintained in a hypophosphorylated and, therefore, active state, thus inhibiting passage through G1 into S phase. Interestingly, at the early stages of *Xenopus *development studied here, E2F is found largely unbound to pRb [61] and, moreover, the *Xenopus *Rb protein is not required for cell cycle regulation or early embryonic development [62]. Additionally, another potential skp2 target, c-myc, is predominantly expressed in the neural crest during early development in *Xenopus *and loss of protein after morpholino injection has little effect on cell proliferation [63]. These observations may explain why loss of skp2 protein does not affect cell cycling at these stages, and indicates that c-myc and E2F are not likely targets for skp2 regulation at this stage.

Absolute levels of skp2 protein are regulated through the cell cycle, being higher in S and G2 phases [34], indicating that the absolute level of skp2 protein is important for its cell cycle function. One characteristic of cancer cells is inappropriate division, and indeed a variety of tumour cells have elevated skp2 levels [38,56]. Moreover, over-expression of skp2 drives entry into S phase, inducing quiescent fibroblasts to replicate their DNA in low serum [30]. Surprisingly, over-expression of skp2 in *Xenopus *embryos results in a small but significant decrease in pH3-staining cells (Figure 3d). However, this decrease is also seen after over-expression of skp2 in which the F box has been deleted and which is severely impaired in its ability to bind to the SCF complex (Figure 3e). Thus, this effect cannot be due to ubiquitin-mediated proteolysis of SCF^skp2 ^targets. However, it is possible that skp2 binding disrupts the function of one of its targets, a positive cell cycle regulator, resulting in slowing of the cell cycle, although in this developmental context it is not clear what such a substrate would be. It has been suggested that in some cases, cdkis at low levels may be required for assembly of cdk complexes, so can act to stimulate the cell cycle, only becoming inhibitory above a certain stoichiometry [64]. Binding by skp2 or F Box skp2 could disrupt this function. However, disruption of such a function of Xic1 in cdk complex assembly could not account for the F Box-dependent requirement for skp2 in primary neurogenesis.

Skp2 levels regulate primary neurogenesis in *Xenopus*; loss of skp2 results in formation of extra neurons (Figure 2) while skp2 over-expression inhibits neurogenesis (Figure 3). *In vitro *studies have shown that Xic1 is a potential target for skp2-mediated ubiquitination and destruction; skp2 can target Xic1 for degradation both *in vitro *and *in vivo *[21] (Figure 6). Supporting this hypothesis, Xic1 is required for primary neurogenesis; in its absence primary neurons do not form, mimicking the effect of skp2 over-expression, while over expression of Xic1 results in supernumery neurons within the usual stripes [22], similar to the pattern seen on injection of skp2 Mo. Interestingly, although Xic1 is a cdk inhibitor, it is not required for neurogenesis at the time of cell cycle exit, downstream of NeuroD. Instead, Xic1 acts at an early stage of neurogenesis in parallel with XNgnr1, the same point at which skp2 acts. Therefore, skp2 may regulate primary neurogenesis by targeting Xic1 for ubiquitin-mediated proteolysis. Most identified substrates of skp2 require cell cycle regulated phosphorylation, generally by cdks, to facilitate skp2 binding. Unusually, ubiquitination by SCF^skp2 ^does not require the phosphorylation of Xic1 [21], indicating that Xic1 levels will be particularly sensitive to absolute levels of skp2 protein.

XNgnr1 over-expression induces extra neurons in neuroectoderm but this is blocked by skp2 over-expression. Can extra Xic1 rescue this loss of ectopic neurons? At the levels injected here, the co-injection of Xic1, skp2 and XNgnr1 frequently results in apoptosis, precluding further investigation. This may be a result of the combined mild pro-apoptotic effects of both XNgnr1 and Xic1 [22,65] when they are co-injected. We would note, however, that Xngnr1 is the endogenous driver of formation of primary neurons in the normal stripes, these are suppressed by over-expression of skp2, and a partial rescue occurs when skp2 and Xic1 are co-injected. We show that Xic1 is required for normal primary neurogenesis, it is degraded by skp2, and a drop in its levels is crucial for over-expressed skp2 to inhibit neurogenesis in the neural plate. Thus, skp2 is perfectly placed to regulate Xic1 levels resulting in control of differentiation of primary neurons *in vivo*.

Over-expression of skp2 results in degradation of a significant proportion of co-expressed Xic1 protein (Figure 6b, c). However, increasing the amount of skp2 further does not result in full degradation of Xic1. In these assays, Xic1 is continually synthesised from micro-injected message and the level of remaining protein may represent an equilibrium between synthesised and degraded Xic1 protein. In addition, at high levels of skp2, it is likely that other components of the Scf E3 ligase complex become limiting. Skp2 mRNA injection has a small but somewhat inconsistent effect on levels of endogenous Xic1 protein in whole embryos that is made up of expression in epidermal, neural and mesodermal tissues. However, by dissecting out the neural plate and neural folds at mid-neurula stage, we can enrich for the population of endogenous Xic1 protein in differentiating primary neurons compared to mesodermal and epidermal Xic1, and when we do this we see that skp2 can target endogenous Xic1 for destruction more efficiently (Figure 6d). Xic1 plays an essential role in muscle differentiation [26]. Interestingly, while skp2 over-expression inhibits neurogenesis, we see that it does not inhibit myotomal muscle differentiation (Figure 6e–h), indicating that it is unable to degrade Xic1 protein in the myotome. This could be due to either lack of other SCF complex members, modification or degradation of skp2 protein, modification of Xic1 or another unknown mechanism. Indeed, endogenously, skp2 is not expressed in muscle so the absence of any required co-factors might not be unexpected.

The mammalian homologue of Xic1, p27Kip1, is also targeted for ubiquitin-mediated proteolysis by the ubiquitin ligase KPC, where its destruction occurs after export from the nucleus [66]. Homologues of its components KPC1 and 2 are found in *Xenopus *and are well conserved (76% and 73% identity with human KPC1 and 2, respectively; C Fiore-Heriche and AP, data not shown). Xic1 destruction in *Xenopus *eggs requires Xic1 to be in the nucleus [67], although it is not known whether later in development there may be an additional cytoplasmic Xic1 destruction pathway. As skp2 is not expressed in the myotome, such a pathway may control Xic1 levels in the developing muscle.

To determine further whether skp2 is acting on neurogenesis via its ability to regulate Xic1 levels, we investigated the effect of co-expression of skp2 and Xic1 on formation of primary neurons (Figure 7). Overexpression of skp2 inhibits neurogenesis while increasing Xic1 levels produces extra neurons: co-injection of the two results in an intermediate phenotype, demonstrating that a balance between levels of skp2 and Xic1 proteins regulates the number of differentiating primary neurons, again indicating that Xic1 is a *bone fide in vivo *substrate of SCF^skp2^. However, it should be noted that Xic1 protein expressed from injected mRNA accumulates to levels far higher than those usually found in embryos (Figure 6), yet rescue is not complete. We favour the possibility that there are unknown protein targets of SCF^skp2^, in addition to Xic1, that are involved in primary neuron differentiation, and that their stability is regulated in parallel with Xic1; levels of Xic1 and this additional target(s) must be restored to fully rescue primary neurogenesis in the presence of elevated skp2 protein. XNgnr1, the proneural protein that drives primary neurogenesis, is unstable but our preliminary data indicate it is unlikely to be a direct target of ubiquitin-mediated proteolysis by SCF^skp2 ^(J Vosper, HB-P and AP, unpublished data). However, other cell cycle regulators known to be targets of SCF^skp2^, such as E2F1 [10] and c-myc [11,12], could play additional roles in regulating neuronal differentiation.

In conclusion, here we demonstrate a role for skp2 in regulating differentiation of primary neurons over and above any role it may have in regulating the cell cycle. Indeed, in *Xenopus*, at these early developmental stages, skp2's role in cell cycle progression appears to be minimal. Skp2 is widely expressed in the developing mouse so it is well placed to play a role in differentiation in mammalian embryos in addition to a role in cell cycle regulation.

Cancer cells divide inappropriately, but equally important is their failure to adopt a fully differentiated character and it is this that results in malignancy. Indeed, cancer may result from a failure to differentiate rather than from cell cycle defects *per se *[68] with cells reverting to a more embryonic phenotype. Skp2 is elevated in a range of tumours [38,56], where our data indicate it may play a separable but complementary role in regulating cell cycle progression and differentiation. We would argue that any direct role in differentiation must be explained to understand the full impact of skp2 upregulation in cancer. In general, molecules that regulate cell cycle exit and differentiation may be crucial targets for cancer therapy, and if we are to make the best use of them we must have a greater understanding of how cell division and differentiation are co-ordinated during development. Identifying molecules with dual functions in these two processes is a good place to start.

## Materials and methods

### Plasmid construction and mutagenesis

To create skp2 without the F-box domain (F Box skp2), two separate fragments flanking the region to be deleted were amplified from skp2 plasmid DNA, using Taq and Vent DNA polymerases. The first fragment (amino acids 1–92 of wild-type skp2) was amplified using primers 5'-CGC AGG GAA TTC GCC ACC ATG CAC AGG AAA CAT CTT CAG GAG ATC TCG-3' and 5'-CGC AGT ACC GGT GAC TGA CTG AGC AAG-3'. The second fragment (amino acids 138–420) was amplified using primers 5'-GTC AGG ACC GGT GAT CTT ACT GGC AAA CAC GTG-3' and 5'-GCA CGC TAG GCC TTC ACA AGT AGT CCT TAT AGT TCA-3'. The PCR products were digested with *Eco*RI/*Age*I (fragment 1) or *Age*I/*Stu*I (fragment 2) and subcloned into the *Eco*RI/*Stu*I sites of pCS2+.

### RNA/morpholino oligonucleotide injections

For generating capped mRNAs, vectors encoding XNgnr1 [23], XNeuroD [24], skp2, F-Box skp2, MAFbx, and ßgal were transcribed *in vitro *(mMessage mMachine Kit, Ambion (Applied Biosystems, Warrington UK). RNAs for XNgnr1 (50 pg), NeuroD (500 pg), skp2 (1 ng), F-Box skp2 (1 ng) or MAFbx (1 ng) were co-injected in a volume of 10 nl along with 100 pg of ßgal mRNA to act as a lineage tracer. Antisense morpholino oligonucleotide skp2 Mo (20–40 ng; 5'-ATG TTT ACC TGT GCA TGT TCA TCA G-3') or a five base-pair mismatch control morpholino (30–40 ng; 5'-ATC TTT AGC TCT GCA TCT TCT TCA G-3') (GeneTools LLC Philomath, Oregon, USA.), were also coinjected with 100 pg of ßgal mRNA.

### *Xenopus *whole-mount *in situ *hybridisation, antibody staining, and *in situ *hybridisation on sections

Embryos were raised until indicated stages, fixed in MEMFA for 1.5 hours, X-gal (5-bromo-4-chloro-3-indolyl β-galactopyranoside) stained and analyzed by whole mount *in situ *hybridisation [69]. Double *in situ *hybridisations were performed according to [70]. Linearised plasmids from Sox3 (Not1/T7), Bluescript N-tubulin (BamH1/T3) [71], pCS2+ (T7 promoter repaired; *Eco*RI/T7) skp2 were used to generate antisense RNA probes by *in vitro *transcription with digoxigenin-11-UTP (Roche, Welwyn Garden City, UK.) and detected using alkaline-phosphatase-conjugated anti-digoxigenin antibodies (Roche). BM purple was used as a substrate (Roche). pH3 immunostaining was carried out as previously described [72] using a 1:1,000 dilution of primary antibody anti-pH3 (TCS Biologicals, Buckingham, UK.) pH3-positive cells were counted within regions of equal surface area on injected and uninjected bilateral halves of each embryo; percent changes were then determined for each embryo as the ratio of injected/uninjected values. Percent values shown represent averaged results. *In situ *hybridisation on tissue sections was carried out as previously described [73]. Alternatively, embryos were post-fixed in MEMFA, embedded in gelatin/albumin and sectioned by Leica VT1000M vibratome, and mounted in 100% glycerol, (60 micron sections; Figure 1h, i). In Figure 1i, staining of deep mesodermal tissues appears light blue due to poor penetrance of the BM purple staining component.

### Western blotting

Protein extracts were prepared as previously described [61]. Skp2 was detected using an affinity purified polyclonal antibody [21], p27Xic1 was detected using an affinity purified polyclonal antibody [21]. Blots were probed with monoclonal anti-tubulin antibody (1: 2,000; Sigma, Gillingham, UK) where appropriate as a loading control. Usually, protein from approximately one embryo equivalent or five dissected neural plates was loaded per lane.

## Competing interests

The author(s) declare that they have no competing interests.

## Authors' contributions

HB-P performed most of the experiments in this paper, with experimental assistance from IH and HW and AP, particularly in analysis of Xic1 protein levels. HRL, LCC and PRY helped HB-P with initial cloning of skp2 and provided essential reagents. HB-P and AP wrote the manuscript. All authors read and approved the final manuscript.

## Supplementary Material

Additional File 1 Categories of nßt expression. Representative examples of embryos displaying each class of nßt expression, as used for quantification. (e) A moderate increase in primary neurons outside the usual stripes (see Figure 5). (g) A moderate increase in primary neurons within the usual stripes (see Figures 4 and 7). Dorsal view with injected side facing rightward. Click here for file
