# Supplementary figures and images for: The E3 ubiquitin ligase skp2 regulates neural differentiation independent from the cell cycle
Source: Neural Dev. 2007 Dec 14;2:27. doi: 10.1186/1749-8104-2-27 (PMC2244796; doi:10.1186/1749-8104-2-27)

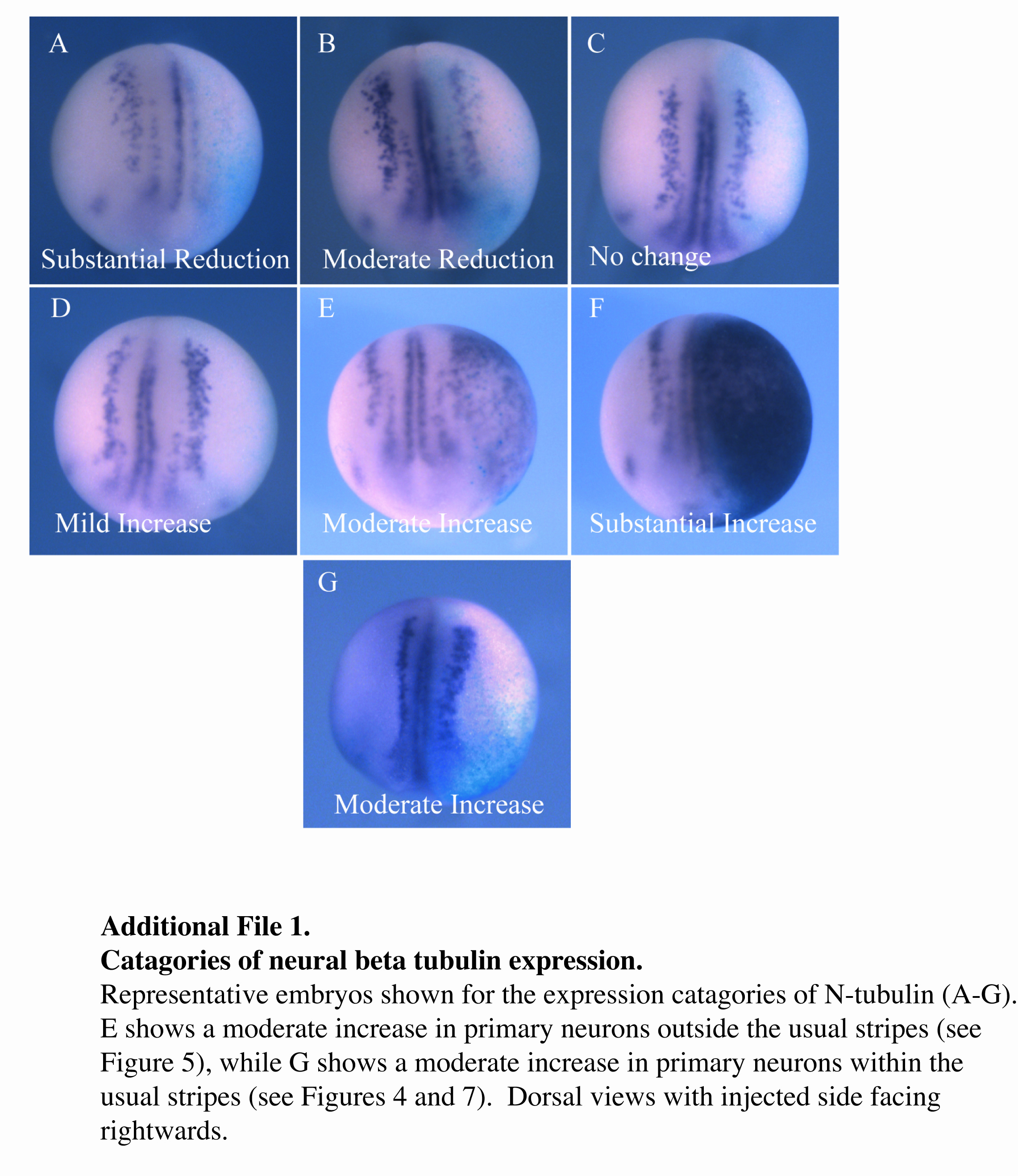

Supplement: Additional File 1 — Categories of nßt expression. Representative examples of embryos displaying each class of nßt expression, as used for quantification. (e) A moderate increase in primary neurons outside the usual stripes (see Figure 5). (g) A moderate increase in primary neurons within the usual stripes (see Figures 4 and 7). Dorsal view with injected side facing rightward. [file 1749-8104-2-27-7-S1.jpeg]
